# Association between Dietary Patterns and the Risk of Depressive Symptoms in the Older Adults in Rural China

**DOI:** 10.3390/nu14173538

**Published:** 2022-08-27

**Authors:** Jing Yan, Qinghan Ren, Hongyan Lin, Qian Liu, Jingzhu Fu, Changqing Sun, Wen Li, Fei Ma, Yun Zhu, Zhenshu Li, Guoquan Zhang, Yue Du, Huan Liu, Xumei Zhang, Yongjie Chen, Guangshun Wang, Guowei Huang

**Affiliations:** 1 Department of Nutrition and Food Science, School of Public Health, Tianjin Medical University, 22 Qixiangtai Road, Heping District, Tianjin 300070, China; 2 Department of Social Medicine and Health Administration, School of Public Health, Tianjin Medical University, 22 Qixiangtai Road, Heping District, Tianjin 300070, China; 3 Tianjin Key Laboratory of Environment, Nutrition and Public Health, 22 Qixiangtai Road, Heping District, Tianjin 300070, China; 4 Neurosurgical Department, Baodi Clinical College, Tianjin Medical University, 8 Guangchuan Road, Baodi District, Tianjin 301899, China; 5 Department of Epidemiology and Biostatistics, School of Public Health, Tianjin Medical University, 22 Qixiangtai Road, Heping District, Tianjin 300070, China; 6 Department of Tumour, Baodi Clinical College, Tianjin Medical University, 8 Guangchuan Road, Baodi District, Tianjin 301899, China

**Keywords:** depression, dietary patterns, inflammatory dietary pattern, older adults, rural areas

## Abstract

Geriatric depression, a chronic condition, has become a substantial burden in rural China. This study aimed to assess the association between dietary patterns and the risk of geriatric depression in rural China. Between March 2018 and June 2019, 3304 participants were recruited for this cross-sectional study in rural Tianjin, China. Principal component analysis was used to determine the major dietary patterns. The associations between dietary patterns and the risk of geriatric depression were assessed using a logistic regression model. Four dietary patterns were identified: vegetables-fruit, animal food, processed food, and milk-egg. The study found that vegetable-fruit (Q2 vs. Q1: *OR* = 0.62, 95% CI: 0.46–0.83; Q3 vs. Q1: *OR* = 0.54, 95% CI: 0.38–0.75; Q4 vs. Q1: *OR* = 0.39, 95% CI: 0.26–0.57) and animal food patterns (Q3 vs. Q1: *OR* = 0.69, 95% CI: 0.50–0.95; Q4 vs. Q1: *OR* = 0.58, 95% CI: 0.41–0.82) were associated with a decreased risk of depression, and inflammatory dietary pattern (Q2 vs. Q1: *OR* = 1.71, 95% CI: 1.23–2.38; Q3 vs. Q1: *OR* = 1.70, 95% CI: 1.22–2.36; Q4 vs. Q1: *OR* = 1.44, 95% CI: 1.03–2.03) was associated with an increased risk of depression. The present findings reinforce the importance of adopting an adequate diet consisting of vegetables, fruit and animal foods, while limiting the intake of pro-inflammatory foods, to decrease the risk of depression.

## 1. Introduction

An estimated 322 million people globally suffer from depression, and the prevalence of geriatric depression is 7%, with trends seemingly increasing yearly [1]. Depression not only causes its more known effects, including persistent sadness, a lack of interest or pleasure in previously rewarding or enjoyable activities, sleep disorders, disturbed appetite, tiredness, and poor concentration, but also causes more severe health effects, such as cardiovascular diseases, stroke, coronary heart disease, gastrointestinal disorders, hypertension, asthma, disability, self-harm, and suicide [2,3]. Thus, it contributes significantly to the global burden of disease. Compared to younger individuals, older adults have a higher prevalence of depression, which has a greater impact on their physical health and cognition [1,4]. In China, the prevalence of depression in older adults ranges from 4% to 26.5%, which may lead to a substantial burden to families and society [5,6]. A previous review reported that the prevalence of depressive symptoms in the older adult population was 22.7%, with 29.2% and 20.5% of these individuals residing in rural and urban China, respectively [5]. This study focuses on older adults in rural China, where the aging population is growing, and residents are of low socioeconomic status.

The current treatments for depressive symptoms include pharmacotherapy, psychotherapy, and lifestyle management [1]. However, in low- and middle-income countries, an estimated 76–85% of people suffering from mental disorders lack access to the corresponding treatment because the treatment and support services for depression are often absent or underdeveloped in these counties [1]. Moreover, despite being the more accessible treatment option, long-term antidepressant treatment may cause significant side effects [7,8]. Growing evidence suggests that certain dietary nutrients (e.g., omega-3 fatty acids, folate, vitamins B and E) or food (e.g., fruit, vegetables, legumes, nuts, fish, and red meat) are associated with depression [7,8,9,10,11,12]; however, complex nutritional interactions and cumulative effects may not be adequately considered when nutrients or foods are studied in isolation [2,4]. Dietary patterns are multifaceted, which makes it difficult to attribute depression to a single nutrient or food group. Therefore, it is important to assess the relationship between dietary patterns as a whole diet and depressive symptoms. Moreover, dietary modifications should be considered a preventative and helpful adjunct to the current line of treatment in place for depression in rural areas.

Previous studies have shown that proinflammatory mechanisms may underpin depressive disorders [2,7,9]. Systemic inflammation and oxidative stress can impair neurotransmitter metabolism and increase neurodegeneration, which may trigger clinical symptoms of depression [7,8,9,10]. This common pathophysiological mechanism underscores the importance of diet in facilitating or impeding chronic inflammatory diseases, such as depression. Some foods, such as red and processed meats, fried foods, snacks, and beverages, are the leading cause of inflammation; however, higher intake of fruit, vegetables, legumes, fish, and whole grain are associated with greater protective effects [13,14,15,16,17]. In fact, recent studies have reported that inflammatory dietary patterns may be associates with an increased incidence of depression [2,4,9]. Therefore, it is imperative to identify modifiable dietary factors early on and to encourage greater adherence to healthy diets to reduce the likelihood of developing depressive disorders.

This study aimed to investigate the potential associations between dietary patterns and the risk of geriatric depression in rural China to provide new information that can be used in prevention and management of this issue.

## 2. Methods

### 2.1. Design and Study Population

**Figure 1:**
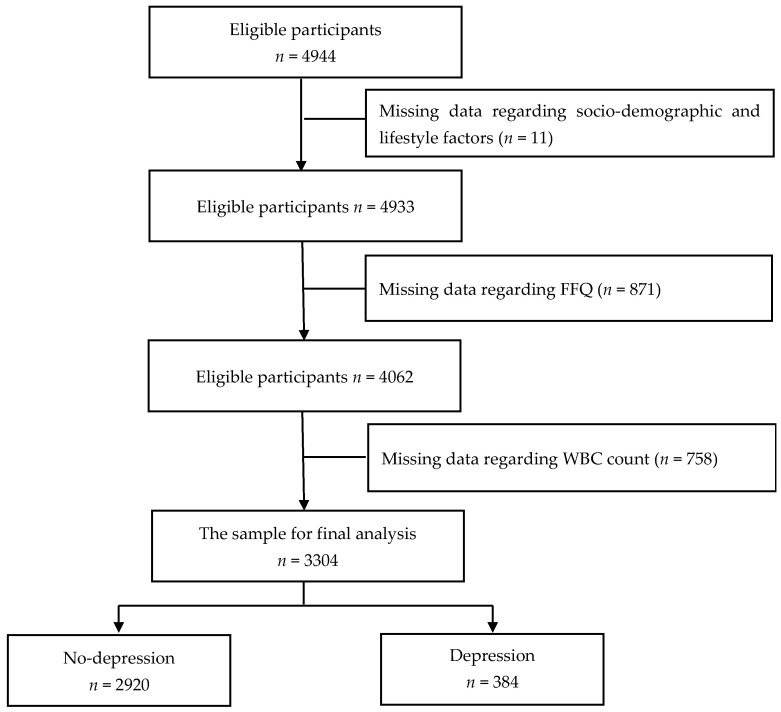
Flow diagram of the enrolment in the study.

This study used data from the Tianjin Elderly Nutrition and Cognition cohort study (Clinical Trial Registration Identifier: ChiCTR 2000034348), which assessed the association between nutrition and cognition in China. A total of 4944 eligible older individuals were recruited between March 2018 and June 2019 from three rural communities in the Baodi District of Tianjin, China. The participants were selected by cluster sampling, that is, all eligible older adults from these three communities were included in this cross-sectional study. The exclusion criteria were as follows: missing data regarding sociodemographic and lifestyle factors (*n* = 11), missing data regarding the food frequency questionnaire (FFQ) (*n* = 871), and missing data regarding white blood cell (WBC) count (*n* = 758). Finally, 3304 participants were included in the final analysis (Figure 1). All participants provided written informed consent, and the study protocol was approved by the ethics committee of Tianjin Medical University, China (no. TMUhMEC2018013).

### 2.2. Assessment of Dietary Patters

The validated quantitative FFQ was used to assess dietary intake. The FFQ uses a seven-point scale, including never or hardly ever eats, <1, 1 time/week, 2–3 times/week, 4–6 times/week, 1 time/day, and ≥2 times/day. The frequency category and quantity (grams/standard portion sizes) of each food were converted to daily intake using the latest available Chinese food composition table [18]. Based on the similarity of nutrients and cooking practices, single food items from the FFQ were classified into 17 predefined food groups (Table 1). Principal component analysis (PCA) was used to determine the major dietary patterns. Reduced-rank regression (RRR) was used to identify the inflammatory dietary pattern. RRR is an extended multivariate linear regression model with the function of dimension reduction, which can explain the maximum variation in a number of response variables. RRR has been widely used in the nutritional epidemiology research to identify dietary patterns based on nutrients or biomarkers (i.e., intermediates between foods and health outcomes) with linear combinations in recent years [13,19]. In this study, both WBC count and neutrophil-to-lymphocyte ratio (NLR) were used as response variables, as these biomarkers are related to inflammation. Food groups with a factor loading greater than 0.3 were major contributors to inflammatory dietary patterns and represented the characteristics of inflammatory diets.

### 2.3. Laboratory Procedures of Inflammatory Markers

Blood samples were drawn in the morning after an overnight fast of approximately 12 h, and the blood samples were drawn and stored at −80 °C to perform several hematology tests. The WBC and percentage of neutrophils and lymphocytes were measured using an automatic blood cell counter (XN-1000).

### 2.4. Assessment of Depressive Symptoms

Face-to-face interviews were conducted in community health service centers. The Chinese version of the Self-rating Depression Scale (SDS), which has been widely used in the Chinese population, was used to measure depressive symptoms [20,21,22]. It is a 20-item self-report scale with a four-point scale (1: “none or a little of the time”; 2: “a small part of the time”; 3: “a lot of time”; and 4: “most of the time”) to evaluate the frequency of symptoms [20,21]. The scale was divided into four categories: (1) pervasive affective disturbances, (2) physiological disturbances, (3) psychomotor disturbances, and (4) psychological disturbances [23]. The standard score is then calculated using the raw score, which is the sum of the scores of the 20 items, multiplied by 1.25 [23,24]. Depressive symptoms were defined by a continuous standard SDS score, with depression being defined as a standard score ≥50 with higher scores reflecting greater symptom severity [24,25,26].

### 2.5. Covariates

The potential confounding factors were age (in years), sex, and education (classified as “<6 years,” “6-years,” and “≥9 years”), household income per month (classified as “<5000 Chinese Yuan (CNY)” and “≥5000 CNY”), employment (classified as “employed or retired” and “unemployed”), living alone, social activities, physical exercise (classified as “Yes” and “No”), sleep duration (classified as “<6 h,” “6–8 h,” “8–10 h” and “≥10 h”), and number of chronic diseases (classified as “0,” “1,” or “≥2”). A general health questionnaire was used to collect this information.

### 2.6. Statistical Analyses

The characteristics of participants with and without depression were compared using the *t*-test for continuous variables and chi-squared test for categorical variables. The Kaiser-Meyer-Olkin (KMO) measure of sampling adequacy and Bartlett’s test of sphericity were used to evaluate the adequacy of correlation matrices with the data. PCA was used to determine the major dietary patterns. The number of factors (dietary patterns) were retained based on an evaluation of the eigenvalues (>1.0), a scree plot, and factor interpretability. The factors were extracted using varimax rotation to maintain an uncorrelated state and improve interpretability. Food groups with a factor loading ≥0.40 were considered as the main contributors to the dietary pattern and represented the character of each pattern. The inflammatory dietary pattern was identified using RRR according to WBC count and NLR. Food groups with a factor loading greater than 0.3 were major contributors to inflammatory dietary patterns and represented the characteristics of inflammatory diets.

Quartiles based on the factor scores were determined for each dietary pattern for further analysis. The baseline characteristics of participants according to quartiles of each dietary pattern were explored using analysis of variance (ANOVA) for continuous variables and the chi-squared test for categorical variables. The SDS scores of participants were calculated across quartiles of each dietary pattern score, and all data were presented as mean ± *SD* and analyzed using ANOVA. The associations between dietary patterns and the risk of geriatric depression were assessed using a logistic regression model.

Statistical analyses were performed using SPSS version 22.0 (SPSS Inc., Chicago, IL, USA) and SAS version 9.3 (SAS Institute Inc., Cary, NC, USA). Significance was set at a two-sided *p* value < 0.05.

## 3. Results

### 3.1. Characteristics According to the Risk of Depression

The final dataset included 3304 older individuals comprised of 1486 (45.0%) males and 1818 (55.0%) females. The mean age of the participants was 67.73 years (standard deviation [*SD*] = 4.88, range: 60–90 years). The overall prevalence of depression in this population was 11.6% (9.0% of male and 13.8% of females). Characteristics associated with a higher risk of depression include being female, lower education level, low household income per month, being unemployed, living alone, lacking social activities, physical inactivity, and longer sleep duration (*p* *<* 0.05) (Table 2).

### 3.2. Factor Loading for Dietary Patterns

The factor-loading matrices for each dietary pattern are presented in Table 3. Both the KMO (0.802) and the Bartlett’s test (*p* < 0.001) showed the adequacy of the correlation matrices among the variables for PCA. Four dietary patterns were identified: (1) labeled vegetable-fruit pattern, which was loaded by high positive factor loading on tubers, fruits, bacteria and algae, legumes, vegetables, and nuts; (2) labeled animal food pattern, which was loaded by high positive factor loading on poultry meat, offal, seafood, and red meat; (3) labeled processed food pattern, which was loaded by high positive factor loading on fried foods, snacks, and pickles; and (4) labeled milk-egg pattern, which was loaded by high positive factor loading on dairy products, eggs, and beverages. These four dietary patterns explained 39.87% of the variance of the food groups, of which vegetables-fruits pattern, animal foods pattern, processed foods pattern and milk-eggs pattern accounted of the total variance in food intakes for 14.71%, 11.19%, 7.43%, and 6.54%, respectively. The eigenvalues of these four major dietary patterns were 3.142, 1.393, 1.179 and 1.064, respectively. The inflammatory dietary pattern, with both WBC count and NLR as response variables, was identified using RRR. For this dietary pattern, higher scores indicated a higher intake of fried foods, snacks, beverages, and pickles. This dietary pattern was labeled the inflammatory dietary pattern and explained 6.23% of the food groups and 0.70% of the response variables.

### 3.3. Associations between Dietary Patterns and Depression

With an increase in the quartiles of the vegetable-fruit pattern, depressive symptoms were found to decrease (*p* < 0.05). The pervasive affective, physiological, psychomotor, and psychological scores showed similar trends (*p* < 0.05). Participants in the highest quartile of animal food patterns had the lowest SDS, physiological, psychomotor, and psychological scores (*p* < 0.05). Moreover, participants in the highest quartile of the milk-egg pattern had the lowest SDS, pervasive affective, psychomotor, and psychological scores (*p* < 0.05). However, participants in the lowest quartile of processed food pattern and inflammatory dietary pattern had the lowest SDS, pervasive affective, physiological, psychomotor, and psychological scores (*p* < 0.05) (Table 4).

The baseline characteristics of the participants according to the quartiles of each dietary pattern are shown in Table S1. The multivariate odds ratios (*ORs*) (95% CIs) for depressive symptoms across the quartiles of each dietary pattern are shown in Table 5. After the final adjustment for possible confounding factors, compared with the first quartile of vegetable-fruit pattern, the other three quartiles were significantly associated with a decreased risk of geriatric depression: the second quartile (*OR*: 0.62, 95% CI: 0.46–0.83, *p* < 0.05), third quartile (*OR*: 0.54, 95% CI: 0.38–0.75, *p* < 0.05), and fourth quartile (*OR*: 0.39, 95% CI: 0.26–0.57, *p* < 0.05). Compared with the first quartile of animal food patterns, ORs in the third and fourth quartiles were 0.69 (95% CI: 0.50–0.95, *p* < 0.05), 0.58 (95% CI: 0.41–0.82, *p* < 0.05), respectively. Compared with the first quartile of inflammatory dietary pattern, the risk estimates of geriatric depression for the second, third, and fourth quartiles were 1.71 (95% CI: 1.23–2.38, *p* < 0.05), 1.70 (95% CI: 1.22–2.36, *p* < 0.05), and 1.44 (95% CI: 1.03–2.03, *p* < 0.05), respectively. However, no significant associations were found between processed food patterns, milk–egg patterns, and depression.

## 4. Discussion

Previous studies have reported that the prevalence of late-life depression was 7% in the world, 17.1% in the population over 75 years old, and 19.47% in Western countries [1,27,28]. In the present study, the prevalence of depressive symptoms was 11.6% among the older adults in rural Tianjin, China. This study identified four dietary patterns (vegetables-fruit, animal food, processed food, and milk-egg) and one inflammatory pattern. This study found that vegetable-fruit and animal food patterns were associated with a decreased risk of depressive symptoms, whereas an inflammatory dietary pattern was associated with an increased risk of depression. Several previous studies reported that a healthy dietary pattern characterized by a high intake of vegetables, fruits, whole grains, olive oil, fish, soy, poultry, and low-fat dairy was associated with a lower risk of depression [2,12,29]. Moreover, an inverse dose-response relationship was found between depression and fruit, nuts, monounsaturated-to-saturated-fatty-acids ratio, and legumes in the Structured Clinical Interview for the Diagnostic and Statistical Manual of Mental Disorders-Fourth Edition [30]. Similar associations for plant foods and depressive symptoms were found in Australia [31]; a diet with a high intake of vegetables, fruit, soy products, mushrooms, and tea was related to lower depressive symptoms in Japan [32], and dietary patterns with a high intake of vegetables, fish, and fruit were related to lower depressive symptoms in Britain [33]. The Mediterranean dietary pattern, which mainly consists of green leafy vegetables, fruit and nuts, legumes, olive oil, cereals, red wine, fish and low dietary intakes of meat products and dairy, has been though to reduce depressive symptoms [7,10]. Several possible reasons have been reported for the effect of healthy dietary patterns on the lower risk of depression. Antioxidant compounds (e.g., vitamins C and E), anti-inflammatory compounds (e.g., choline, folate and curcumin), and nutrients that influence the gut microbiota (e.g., fiber and polyphenols) found in vegetables and fruits can reduce the risk of depression [34,35,36]. In addition, nuts containing sufficient amounts of tryptophan, vitamin B, flavonoids and polyunsaturated fatty acids have antidepression effects [9,20,37]. Zinc, iron, and selenium rich in meat and offal are cofactors for antioxidant systems, which decrease the risk of depression [9,34,38]. Tryptophan, which is found mostly in chicken, tuna, nuts, bananas, milk, and cheese, is an important component of neuroactive molecules and is converted to serotonin, which has antidepressant effects [9,10,11,12]. Omega-3 fatty acids and polyunsaturated fatty acids (e.g., docosahexaenoic acid, eicosapentaenoic acid and alpha-linoleic acid), which have anti-inflammatory properties and are abundant in fish, have also been found to reduce the risk of depression [7,12,36].

A pro-inflammatory diet was associated with an increased risk of depression symptoms, and the risk of depression was reduced by an anti-inflammatory diet such as a Mediterranean diet in longitudinal observational cohorts and meta-analyses [10,14,35,39]. However, these findings are inconsistent. A previous study did not find a longitudinal association between inflammatory dietary patterns and a high risk of depressive symptoms in older Italian individuals [13]. Moreover, several studies showed the higher dietary inflammatory index (DII) related with higher risk of depressive symptoms [15,40]. The potential mechanisms include pro-inflammatory nutrients in the pro-inflammatory foods that activate the innate immune system and then cause low-grade inflammation and mental health disorders, which could affect depressive symptoms by affecting neuronal function and synaptic plasticity [2,3,14]. Previous studies have reported that a high intake of pro-inflammatory foods was associated with increased concentrations of inflammatory cytokines (e.g., C-reactive protein, interleukin-6 and interleukin-17) and oxidative stress markers (e.g., malondialdehyde and 8-F2-isoprostanes), impaired neurogenesis and lower level of brain-derived neurotrophic factor (BDNF), which are all correlated with mental health disorders. In contrast, a high consumption of anti-inflammatory foods was associated with reduced concentrations of inflammatory and oxidative stress markers, and a beneficial effect on BDNF expression against depressive symptoms [9,13,14].

In contrast, there was no relationship between processed food patterns, milk–egg patterns, and depression, which is consistent with two previous studies [41,42]. In fact, unhealthy dietary patterns characterized by a high intake of fast food, sweet snacks, beverages, dairy products, whole grains, coffee, nuts, and eggs were not related to depression [41]. Similarly, there was no association between depression and a dietary pattern characterized by a high intake of fruit juice, vegetables, nuts, grains, pizza, pasta dishes, chocolates and sweets, snacks, processed meat, or high-fat dairy [42]. In line with this, three meta-analyses showed that unhealthy dietary patterns were associated with an increased risk of depressive symptoms of depression [7,12,43], and a longitudinal study showed that high consumption of processed foods was frequently associated with increases in depressive symptoms [33].

To our knowledge, this is the first study to have investigated the association between dietary patterns and depression symptoms in rural older Chinese individuals and the association between inflammatory dietary patterns and depression in older Chinese. Another strength of this study was that adjusting for several sociodemographic variables reduced the likelihood of confounding. However, some potential limitations of this study warrant consideration. First, recall bias was possible because data on dietary intake were collected using the FFQ. Second, although several covariates were involved in this study, more confounding factors (e.g., family support, smoking, marriage, cardiovascular, dental, clinical, pharmacological status and other biochemical parameters) need to be evaluated in future studies. Finally, the present study used WBC count and NLR as response variables to identify the inflammatory dietary pattern, which may not be good markers. In subsequent follow-ups, other inflammatory cytokines should be considered and included in the analysis of inflammatory dietary pattern.

## 5. Conclusions

Geriatric depression is a serious mental health disorder in rural China. This study on the association between dietary patterns and depressive symptoms showed that the vegetables-fruit and animal food patterns were associated with a decreased risk of depression, and inflammatory dietary pattern was associated with an increased risk of depression. However, further investigation is required to verify the underlying mechanisms.

## Figures and Tables

**Table 1:** Food grouping using in the dietary pattern analysis.

Food Groups	Food Items
Tubers	Sweet potato, yam, taro, potato and products
Fruits	Stone fruits, pome fruits, melon fruits, berry fruits, citrus fruits and grape
Bacteria and algae	Mushroom, fungi, white fungus, kelp, seaweed and other algae
Legumes	Soybeans, other beans and soy products
Vegetables	Leaf vegetables, root vegetables, stem vegetables, melon and eggplant vegetables and flower vegetables
Nuts	Nuts, walnuts, peanuts, sesame, other nuts and products
Poultry meat	Chicken, duck, goose
Offal	Heart, liver, kidney, lung
Seafood	Fish and shellfish
Red meat	Pork, mutton, lamb, beef
Fried foods	Fried dough twist, deep-fried cake, fried dough sticks and instant noodles
Snacks	Ice cream, cake, pastry, snacks, butter products, candied fruits, confections and added sugar
Pickles	Pickles
Grains	Whole grains and refined grains
Dairy products	Milk, yoghurt and other dairy products
Eggs	Eggs, duck eggs and preserved eggs
Beverages	Fruit- and vegetable juices, soft beverages, tea, red wine, white spirits, beer and other alcoholic beverages

**Table 2:** Characteristics of participants with and without depression [Mean *± SD*/*n* (%)].

Characteristics		Total (*n* = 3304)	Depression		
			Yes (*n* = 384)	No (*n* = 2920)	*p* ^a^
Age (years)		67.73 ± 4.88	67.77 ± 4.85	67.72 ± 4.89	0.864
Sex	Male	1486 (44.98%)	134 (9.02%)	1352 (90.98%)	<0.001
	Female	1818 (55.02%)	250 (13.75%)	1568 (86.25%)	
Education (years)	<6	792 (23.97%)	125 (15.78%)	667 (84.22%)	<0.001
	6-	1108 (33.54%)	134 (12.09%)	974 (87.91%)	
	≥9	1404 (42.49%)	125 (8.90%)	1279 (91.10%)	
Household income per month (CNY)	<5000	2782 (84.20%)	358 (12.87%)	2424 (87.13%)	<0.001
	≥5000	522 (15.80%)	26 (4.98%)	496 (95.02%)	
Employment	Employed or retired	1140 (34.50%)	88 (7.72%)	1052 (92.28%)	<0.001
	Unemployed	2164 (65.50%)	296 (13.68%)	1868 (86.32%)	
Living alone	Yes	245 (7.42%)	45 (18.37%)	200 (81.63%)	0.001
	No	3059 (92.58%)	339 (11.08%)	2720 (88.92%)	
Social activities	Yes	1063 (32.17%)	90 (8.47%)	973 (91.53%)	<0.001
	No	2241 (67.83%)	294 (13.12%)	1947 (86.88%)	
Physical exercises	Yes	2913 (88.17%)	304 (10.44%)	2609 (89.56%)	<0.001
	No	391 (11.83%)	80 (20.46%)	311 (79.54%)	
Sleep duration (h)	<6	58 (1.76%)	6 (10.34%)	52 (89.66%)	0.016
	6-	944 (28.57%)	101 (10.70%)	843 (89.30%)	
	8-	1986 (60.11%)	223 (11.23%)	1763 (88.77%)	
	≥10	316 (9.56%)	54 (17.09%)	262 (82.91%)	
No. chronic diseases	0	1576 (47.70%)	190 (12.06%)	1386 (87.94%)	0.059
	1	1284 (38.86%)	131 (10.20%)	1153 (89.80%)	
	≥2	444 (13.44%)	63 (14.19%)	381 (85.81%)	

CNY: Chinese Yuan; ^a^ analysis using an independent-sample *t*-test and chi-squared test; participants were divided into two groups according to self-rating depression scale scores: <50 for no depression and ≥50 for depression.

**Table 3:** Factor loadings of food groups for dietary patterns.

Food Groups	PCA				RRR
	Vegetables-Fruit Pattern	Animal Food Pattern	Processed Food Pattern	Milk-Egg Pattern	Inflammatory Dietary Pattern
Tubers	0.606	0.114	−0.037	−0.084	−0.232
Fruits	0.596	0.039	0.031	0.239	0.297
Bacteria and algae	0.595	0.103	0.196	−0.120	−0.181
Legumes	0.573	0.257	0.191	−0.117	−0.245
Vegetables	0.572	0.153	−0.203	0.051	−0.209
Nuts	0.560	−0.007	0.124	0.153	−0.033
Poultry meat	0.157	0.771	0.062	0.040	−0.145
Offal	−0.074	0.758	0.128	0.014	0.143
Seafood	0.324	0.615	−0.032	0.013	−0.099
Red meat	0.360	0.439	−0.084	0.045	−0.202
Fried foods	−0.011	0.130	0.690	0.060	0.516
Snacks	0.002	0.111	0.602	0.364	0.372
Pickles	0.296	0.046	0.406	−0.300	0.305
Grains	0.038	−0.075	0.256	−0.120	−0.009
Dairy products	0.319	−0.066	0.002	0.601	−0.086
Eggs	−0.030	0.078	0.120	0.442	0.039
Beverages	−0.023	0.003	−0.135	0.440	0.348
Explained variation in food groups (%)	14.71	11.19	7.43	6.54	6.23
Explained variation in WBC count and NLR (%)	--	--	--	--	0.70

PCA, principal component analysis; RRR, reduced rank regression; WBC, white blood cell; NLR, neutrophil-to-lymphocyte ratio.

**Table 4:** Depression self-rating scale score according to categories of dietary patterns (*n* = 3304, Mean ± *SD*).

Dietary Patterns	Quartiles of Dietary Patterns				*p*
	Q1	Q2	Q3	Q4	
Vegetables-fruits pattern					
*n*	826	826	826	826	
SDS score	41.04 ± 9.27	38.56 ± 8.97	36.32 ± 9.06	34.96 ± 8.35	<0.001
Pervasive affective	2.68 ± 1.07	2.53 ± 1.00	2.42 ± 0.90	2.40 ± 0.85	<0.001
Physiological	13.31 ± 3.01	12.69 ± 3.25	12.11 ± 3.28	11.89 ± 3.09	<0.001
Psychomotor	3.40 ± 1.29	3.10 ± 1.20	2.91 ± 1.20	2.87 ± 1.21	<0.001
Psychological	13.45 ± 4.06	12.52 ± 4.10	11.61 ± 3.92	10.81 ± 3.60	<0.001
Animal foods pattern					
*n*	826	826	826	826	
SDS score	38.23 ± 9.38	39.49 ± 9.58	37.32 ± 9.12	35.85 ± 8.33	<0.001
Pervasive affective	2.52 ± 1.00	2.63 ± 1.04	2.43 ± 0.86	2.46 ± 0.95	<0.001
Physiological	12.76 ± 3.26	12.94 ± 3.12	12.29 ± 3.21	12.01 ± 3.15	<0.001
Psychomotor	3.14 ± 1.34	3.20 ± 1.26	3.00 ± 1.17	2.94 ± 1.19	<0.001
Psychological	12.17 ± 4.11	12.82 ± 4.25	12.13 ± 4.06	11.28 ± 3.58	<0.001
Processed foods pattern					
*n*	826	826	826	826	
SDS score	35.39 ± 9.25	39.06 ± 9.57	37.99 ± 8.63	38.44 ± 8.94	<0.001
Pervasive affective	2.43 ± 0.91	2.56 ± 1.01	2.49 ± 0.93	2.54 ± 1.01	0.033
Physiological	11.97 ± 3.33	12.82 ± 3.21	12.50 ± 2.94	12.72 ± 3.28	<0.001
Psychomotor	2.86 ± 1.25	3.15 ± 1.24	3.08 ± 1.19	3.19 ± 1.27	<0.001
Psychological	11.05 ± 3.85	12.72 ± 4.18	12.33 ± 4.01	12.30 ± 3.94	<0.001
Milk-eggs pattern					
*n*	826	826	826	826	
SDS score	37.57 ± 9.23	39.08 ± 9.67	37.40 ± 9.01	36.84 ± 8.77	<0.001
Pervasive affective	2.50 ± 0.96	2.59 ± 1.04	2.52 ± 0.97	2.42 ± 0.88	0.005
Physiological	12.38 ± 3.14	12.83 ± 3.29	12.40 ± 3.14	12.39 ± 3.24	0.010
Psychomotor	3.07 ± 1.23	3.14 ± 1.23	3.05 ± 1.24	3.02 ± 1.28	0.217
Psychological	12.10 ± 4.10	12.71 ± 4.21	11.95 ± 3.94	11.64 ± 3.85	<0.001
Inflammatory dietary pattern					
*n*	831	824	824	825	
SDS score	35.43 ± 8.91	38.17 ± 9.53	38.70 ± 9.18	38.60 ± 8.82	<0.001
Pervasive affective	2.39 ± 0.85	2.50 ± 0.94	2.55 ± 1.02	2.59 ± 1.04	<0.001
Physiological	12.00 ± 3.30	12.58 ± 3.30	12.67 ± 3.05	12.76 ± 3.12	<0.001
Psychomotor	2.87 ± 1.23	3.11 ± 1.25	3.13 ± 1.21	3.17 ± 1.27	<0.001
Psychological	11.09 ± 3.82	12.35 ± 4.21	12.61 ± 4.07	12.35 ± 3.90	<0.001

Q, quartile; SDS: the Self-Rating Depression Scale.

**Table 5:** Associations between each dietary pattern quartiles and depression [*OR* (95% CI)].

Dietary Patterns	Quartiles of Dietary Patterns			
	Q1	Q2	Q3	Q4
Vegetables-fruits pattern				
*n*	826	826	826	826
Model 1 ^a^	1.00	0.58 (0.44,0.76) *	0.46 (0.34,0.61) *	0.31 (0.23,0.43) *
Model 2 ^b^	1.00	0.60 (0.44,0.80) *	0.47 (0.34,0.65) *	0.31 (0.21,0.45) *
Model 3 ^c^	1.00	0.62 (0.46,0.83) *	0.54 (0.38,0.75) *	0.39 (0.26,0.57) *
Animal foods pattern				
*n*	826	826	826	826
Model 1 ^a^	1.00	1.26 (0.96,1.67)	0.73 (0.54,0.99) *	0.54 (0.39,0.75) *
Model 2 ^b^	1.00	0.94 (0.70,1.26)	0.65 (0.47,0.90) *	0.51 (0.37,0.72) *
Model 3 ^c^	1.00	0.94 (0.69,1.26)	0.69 (0.50,0.95) *	0.58 (0.41,0.82) *
Processed foods pattern				
*n*	826	826	826	826
Model 1 ^a^	1.00	1.51 (1.11,2.04) *	1.08 (0.79,1.44)	1.33 (0.98,1.81)
Model 2 ^b^	1.00	0.86 (0.62,1.21)	0.82 (0.59,1.15)	1.12 (0.81,1.55)
Model 3 ^c^	1.00	0.92 (0.65,1.28)	0.83 (0.59,1.17)	1.11 (0.80,1.54)
Milk-eggs pattern				
*n*	826	826	826	826
Model 1 ^a^	1.00	1.35 (1.01,1.81) *	0.99 (0.73,1.34)	0.82 (0.60,1.13)
Model 2 ^b^	1.00	0.87 (0.63,1.22)	0.79 (0.58,1.09)	0.77 (0.56,1.07)
Model 3 ^c^	1.00	0.93 (0.67,1.30)	0.84 (0.61,1.17)	0.84 (0.60,1.18)
Inflammatory dietary pattern				
*n*	831	824	824	825
Model 1 ^a^	1.00	1.85 (1.34,2.55) *	1.87 (1.35,2.58) *	1.63 (1.17,2.27) *
Model 2 ^d^	1.00	1.71 (1.23,2.38) *	1.70 (1.22,2.36) *	1.44 (1.03,2.03) *

Q, quartile; *OR*, odds ratio; CI, confidence interval. Analysis using logistic regression model. * *p* < 0.05. ^a^ Crude model. ^b^ Adjusted for the scores of vegetarian pattern, animal food pattern, processed food pattern, and milk-egg pattern. ^c^ Further adjusted for age, sex, education, household income per month, employment, living alone, social activities, physical exercise, sleep duration, no. chronic diseases. ^d^ Adjusted for age, sex, education, household income per month, employment, living alone, social activities, physical exercise, sleep duration, No. chronic diseases.

## Data Availability

The data presented in this study are available upon reasonable request from the corresponding author. The data are not publicly available due to data safety reasons.

## Supplementary Materials

The following supporting information can be downloaded at: https://www.mdpi.com/article/10.3390/nu14173538/s1, Table S1: Characteristics of the study participants by quartile categories of dietary pattern scores

## Abbreviations

The following abbreviations are used in this manuscript:

ANOVA	analysis of variance
BDNF	brain-derived neurotrophic factor
CI	confidence interval
CNY	Chinese Yuan
FFQ	food frequency questionnaire
KMO	Kaiser-Meyer-Olkin
NLR	neutrophil to lymphocyte ratio
OR	odds ratio
PCA	principal component analysis
RRR	reduced-rank regression
SD	standard deviation
SDS	self-rating depression scale
WBC	white blood cell
