# Supplementary material for: Association between Dietary Patterns and the Risk of Depressive Symptoms in the Older Adults in Rural China
Source: Nutrients. 2022 Aug 27;14(17):3538. doi: 10.3390/nu14173538 (PMC9460296; doi:10.3390/nu14173538)
Supplement: Supplementary file 1 [file nutrients-14-03538-s001.zip › nutrients-1856536-supplementary.pdf]

Table S1. Characteristics of the study participants by quartile categories of dietary pattern scores [*n*=3304, Mean ± *SD*/*n* (%)]

Characteristics	Vegetables-fruits pattern					Animal foods pattern					Processed foods pattern					Milk-eggs pattern					Inflammatory dietary pattern				
	Q1(*n* = 826)	Q2(*n* = 826)	Q3(*n* = 826)	Q4(*n* = 826)	*p*	Q1(*n* = 826)	Q2(*n* = 826)	Q3(*n* = 826)	Q4(*n* = 826)	*p*	Q1(*n* = 826)	Q2(*n* = 826)	Q3(*n* = 826)	Q4(*n* = 826)	*p*	Q1(*n* = 826)	Q2(*n* = 826)	Q3(*n* = 826)	Q4(*n* = 826)	*p*	Q1(*n* = 831)	Q2(*n* = 824)	Q3(*n* = 824)	Q4(*n* = 825)	*p*
Age (years)	67.63 ± 4.85	67.82 ± 5.03	67.79 ± 4.87	67.67 ± 4.79	0.823	68.22 ± 4.99	67.68 ± 4.88	67.70 ± 4.84	67.32 ± 4.79	0.003	67.40 ± 4.69	67.39 ± 4.80	67.81 ± 4.79	68.31 ± 5.19	< 0.001	67.10 ± 4.49	67.165 ± 4.62	67.66 ± 4.84	69.00 ± 5.31	< 0.001	67.44 ± 4.73	67.65 ± 4.88	67.65 ± 4.72	68.17 ± 5.17	0.018
Sex					0.003					< 0.001					< 0.001					0.004					1.000
Male	350(23.6%)	346(23.3%)	378(25.4%)	412(27.7%)		293(19.7%)	300(20.2%)	408(27.5%)	485(32.6%)		364(24.5%)	344(23.1%)	349(23.5%)	429(28.9%)		352(23.7%)	347(23.4%)	374(25.2%)	413(27.8%)		375(25.2%)	370(24.9%)	370(24.9%)	371(25.0%)	
Female	476(26.2%)	480(26.4%)	448(24.6%)	414(22.8%)		533(29.3%)	526(28.9%)	418(23.0%)	341(18.8%)		462(25.4%)	482(26.5%)	477(26.2%)	397(21.8%)		474(26.1%)	479(26.3%)	452(24.9%)	413(22.7%)		456(25.0%)	451(25.0%)	454(25.0%)	454(25.0%)	
Education (years)					< 0.001					< 0.001					0.001					< 0.001					< 0.001
< 6	249(31.4%)	220(27.8%)	164(20.7%)	159(20.1%)		222(28.0%)	243(30.7%)	165(20.8%)	162(20.5%)		182(23.0%)	222(28.0%)	192(24.2%)	196(24.7%)		229(28.9%)	229(28.9%)	176(22.2%)	157(19.9%)		167(19.8%)	180(22.7%)	222(28.0%)	233(29.4%)	
6 -	316(28.5%)	282(25.5%)	272(24.5%)	238(21.5%)		298(26.9%)	298(26.9%)	266(24.0%)	246(22.2%)		240(21.7%)	275(24.8%)	301(27.2%)	292(26.4%)		264(23.8%)	304(27.4%)	280(25.3%)	260(23.5%)		240(21.7%)	272(24.5%)	300(27.1%)	296(26.7%)	
⩾ 9	261(18.6%)	324(23.1%)	390(27.8%)	429(30.6%)		306(21.8%)	285(20.3%)	395(28.1%)	418(29.8%)		404(28.8%)	329(23.4%)	333(23.7%)	338(24.1%)		333(23.7%)	293(20.9%)	370(26.4%)	408(29.1%)		434(30.9%)	372(26.5%)	202(21.5%)	296(21.1%)	
Household income per month (CNY)					< 0.001					< 0.001					< 0.001					0.036					< 0.001
< 5000	747(26.9%)	728(26.2%)	684(24.6%)	623(22.4%)		713(25.6%)	742(26.7%)	673(24.2%)	654(23.5%)		658(23.7%)	691(24.8%)	700(25.2%)	733(26.3%)		688(24.7%)	722(26.0%)	687(24.7%)	685(24.6%)		625(22.5%)	698(25.1%)	721(25.9%)	738(26.5%)	
⩾ 5000	79(15.1%)	98(18.8%)	142(27.2%)	203(38.9%)		113(21.6%)	84(16.1%)	153(29.3%)	172(33.0%)		168(32.2%)	135(25.9%)	126(24.1%)	93(17.8%)		138(26.4%)	104(19.9%)	139(26.6%)	141(27.0%)		206(39.5%)	126(24.1%)	103(19.7%)	87(16.7%)	
Employment					< 0.001					< 0.001					< 0.001					0.001					< 0.001
Employed or retired	241(21.1%)	230(20.2%)	296(26.0%)	373(32.7%)		268(23.5%)	245(21.5%)	289(25.4%)	338(29.6%)		329(28.9%)	313(27.55%)	267(23.4%)	231(20.3%)		251(22.0%)	283(24.8%)	279(24.5%)	327(28.7%)		356(31.2%)	283(24.8%)	283(24.8%)	218(19.1%)	
Unemployed	585(27.0%)	596(27.5%)	530(24.5%)	453(20.9%)		558(25.8%)	581(26.8%)	537(24.8%)	488(22.6%)		497(23.0%)	513(23.7%)	559(25.8%)	595(27.5%)		575(26.6%)	543(25.1%)	547(25.3%)	499(23.1%)		475(22.0%)	541(25.0%)	541(25.0%)	607(28.0%)	

Living alone					0.003					1.000					0.511					0.103					0.573
Yes	72(29.4%)	69(28.2%)	67(27.3%)	37(15.1%)		61(24.9%)	61(24.9%)	62(25.3%)	61(24.9%)		54(22.0%)	58(23.7%)	69(28.2%)	64(26.1%)		50(20.4%)	54(22.0%)	70(28.6%)	71(29.0%)		53(21.6%)	65(26.5%)	66(26.9%)	61(24.9%)	
No	754(24.6%)	757(24.7%)	759(24.8%)	789(25.8%)		765(25.0%)	765(25.0%)	764(25.0%)	765(25.0%)		772(25.2%)	768(25.1%)	757(24.7%)	762(24.9%)		776(25.4%)	772(25.2%)	756(24.7%)	755(24.7%)		778(25.4%)	759(24.8%)	758(24.8%)	764(25.0%)	
Social activities					< 0.001					< 0.001					0.266					0.008					0.029
Yes	226(21.3%)	243(22.9%)	290(27.3%)	304(28.6%)		274(25.8%)	229(21.5%)	235(22.1%)	325(30.6%)		278(26.2%)	246(23.1%)	261(24.6%)	278(26.2%)		237(22.3%)	257(24.2%)	268(25.2%)	301(28.3%)		295(27.8%)	236(22.2%)	269(25.3%)	263(24.7%)	
No	600(26.8%)	583(26.0%)	536(23.9%)	522(23.3%)		552(24.6%)	597(26.6%)	591(26.4%)	501(22.4%)		548(24.5%)	580(25.9%)	565(25.2%)	548(24.5%)		589(26.3%)	569(25.4%)	558(24.9%)	525(23.4%)		536(23.9%)	588(26.2%)	555(24.8%)	562(25.1%)	
Physical exercise					< 0.001					0.159					0.347					0.107					0.654
Yes	698(24.0%)	716(24.6%)	747(25.6%)	752(25.8%)		733(25.2%)	710(24.4%)	735(25.2%)	735(25.2%)		739(25.4%)	720(24.7%)	734(25.2%)	720(24.7%)		723(24.8%)	720(24.7%)	722(24.8%)	748(25.7%)		742(25.5%)	727(25.0%)	723(24.8%)	721(24.8%)	
No	128(32.7%)	110(28.1%)	79(20.2%)	74(18.9%)		93(23.5%)	116(29.7%)	91(23.3%)	91(23.3%)		87(22.3%)	106(27.1%)	92(23.5%)	106(27.1%)		103(26.3%)	106(27.1%)	104(26.6%)	78(19.9%)		89(21.2%)	97(24.8%)	101(25.8%)	114(26.6%)	
Sleep duration (h)					0.002					0.008					< 0.001					0.158					< 0.001
< 6	10(17.2%)	14(24.1%)	17(29.3%)	17(29.3%)		21(36.2%)	11(19.0%)	13(22.4%)	13(22.4%)		10(17.2%)	10(17.2%)	19(32.8%)	19(32.8%)		20(34.5%)	8(13.8%)	11(19.0%)	19(32.8%)		15(25.9%)	7(12.1%)	21(36.2%)	15(25.9%)	
6 -	224(23.7%)	219(23.2%)	239(25.3%)	262(27.8%)		229(24.3%)	209(22.1%)	242(25.6%)	264(28.0%)		289(30.6%)	236(25.0%)	211(22.4%)	208(22.0%)		248(26.3%)	238(25.2%)	242(25.6%)	216(22.9%)		286(30.3%)	242(25.6%)	213(23.6%)	203(21.5%)	
8 -	483(24.3%)	512(25.8%)	505(25.4%)	486(24.5%)		479(24.1%)	520(26.2%)	508(25.6%)	479(24.1%)		471(23.7%)	498(25.1%)	520(26.2%)	497(25.0%)		491(24.7%)	500(25.2%)	486(24.5%)	509(25.6%)		472(23.8%)	510(25.7%)	501(25.2%)	503(25.3%)	
⩾ 10	109(34.5%)	81(25.6%)	65(20.6%)	61(19.3%)		97(30.7%)	86(27.2%)	63(19.9%)	70(22.2%)		56(17.7%)	82(25.9%)	76(24.1%)	102(32.3%)		67(21.2%)	80(25.3%)	87(27.5%)	82(25.9%)		58(18.4%)	65(20.6%)	89(28.2%)	104(32.9%)	
No. chronic diseases					0.232					0.022					0.026					0.099					< 0.001
0	418(26.5%)	395(25.1%)	391(24.8%)	372(23.6%)		386(24.5%)	418(26.5%)	399(25.3%)	373(23.7%)		382(24.2%)	387(24.6%)	373(23.7%)	434(27.5%)		374(23.7%)	395(25.1%)	422(26.8%)	385(24.4%)		351(22.3%)	384(24.4%)	403(25.6%)	438(27.8%)	
1	296(23.1%)	320(24.9%)	318(24.8%)	350(27.3%)		321(25.0%)	302(23.5%)	302(23.5%)	359(28.0%)		319(24.8%)	324(25.2%)	337(26.2%)	304(23.7%)		332(25.9%)	307(23.9%)	313(24.4%)	332(25.9%)		336(26.2%)	337(26.2%)	309(24.1%)	302(23.5%)	
⩾ 2	112(25.2%)	111(25.0%)	117(26.4%)	104(23.4%)		119(26.8%)	106(23.9%)	125(28.2%)	94(21.2%)		125(28.2%)	115(25.9%)	116(26.1%)	88(19.8%)		120(27.0%)	124(27.9%)	91(20.5%)	109(24.5%)		144(32.4%)	103(23.2%)	112(25.2%)	85(19.1%)	

CNY: Chinese Yuan; Analysis using independent-sample *t*-test and chi-squared test.
